# A New Combination

**Published:** 1884-03

**Authors:** 


### EDITORIAL.

### A New Combination.

Prof. Dorrance, of the Dental College of the University of Michigan, mounts artificial teeth on an aluminium base, attached with Watts' metal instead of rubber. The work presents a beautiful appearance and will doubtless be well suited to many cases.

The method of constructing the work is by the same process substantially, as that in making cast plates, except that the aluminium plate is first stamped, and then with the teeth adjusted upon it, is waxed up for the attachment and invested for casting, after which it is finished in the ordinary way. There is no patent on it.

Dr. D. will report further when more experience is had with it in the mouth. We will await the result with interest.

